# Prospective Artificial Intelligence to Dissect the Dengue Immune Response and Discover Therapeutics

**DOI:** 10.3389/fimmu.2021.574411

**Published:** 2021-06-15

**Authors:** Eriberto N. Natali, Lmar M. Babrak, Enkelejda Miho

**Affiliations:** ^1^ Institute of Medical Engineering and Medical Informatics, School of Life Sciences, University of Applied Sciences and Arts Northwestern Switzerland FHNW, Muttenz, Switzerland; ^2^ SIB Swiss Institute of Bioinformatics, Lausanne, Switzerland; ^3^ aiNET GmbH, Basel, Switzerland

**Keywords:** artificial intelligence, dengue, antibody discovery, B-cell receptor, immune repertoire, virus, immunotherapy, machine learning

## Abstract

Dengue virus (DENV) poses a serious threat to global health as the causative agent of dengue fever. The virus is endemic in more than 128 countries resulting in approximately 390 million infection cases each year. Currently, there is no approved therapeutic for treatment nor a fully efficacious vaccine. The development of therapeutics is confounded and hampered by the complexity of the immune response to DENV, in particular to sequential infection with different DENV serotypes (DENV1–5). Researchers have shown that the DENV envelope (E) antigen is primarily responsible for the interaction and subsequent invasion of host cells for all serotypes and can elicit neutralizing antibodies in humans. The advent of high-throughput sequencing and the rapid advancements in computational analysis of complex data, has provided tools for the deconvolution of the DENV immune response. Several types of complex statistical analyses, machine learning models and complex visualizations can be applied to begin answering questions about the B- and T-cell immune responses to multiple infections, antibody-dependent enhancement, identification of novel therapeutics and advance vaccine research.

## Introduction

Dengue virus (DENV) is a member of the *Flaviviridae* family and the etiologic agent of dengue fever. Five serotypes have been reported (DENV1 - DENV5), with the fifth limited only to one outbreak in Sarawak, Malaysia (1, 2). DENV is endemic in 128 countries encompassing Africa, the Americas, the Eastern Mediterranean, South-East Asia and the Western Pacific, and causes an estimated 390 million cases each year, of which 96 million manifest clinically (3). Its global incidence has grown dramatically in the recent decade and now poses a serious threat to public health, especially in Asia, which shoulders 70% of the burden (4). The epicenter of dengue infection is in the Indian subcontinent where the climate and the environment make it difficult to contain infection (5). Due to the increase of travel to low- and middle-income endemic countries, dengue fever is spreading to European countries. Worldwide, it is estimated that approximately 500,000 patients require hospitalization and 12,500 of those dying due to dengue annually (3). The burden of dengue has a great economic impact on countries, particularly developing countries, generating an estimated total annual global cost of 8-9 billion US dollars (6).

## Current State of Vaccine and Therapeutic Development

The main vector for viral transmission of dengue is the female *Aedes aegypti* and *albopictus* mosquitos (7). The mosquito becomes infected by biting an infected individual and after a week the mosquito can bite a healthy person and spread the virus. The virus cannot spread person to person. In humans the primary infection leads to largely asymptomatic cases, but in some cases, can cause non-specific flu-like symptoms (e.g., severe headache, nausea) followed by spontaneous recovery and lifelong serotype-specific immunization (8). Reinfection by a heterologous serotype of dengue can lead to severe and fatal disease such as dengue hemorrhagic fever (DHF). It is currently believed that the dramatic response to heterologous infection is caused by antibody-dependent enhancement (ADE) (8). ADE is caused by an increase of viral particles into hosts cells due to non-specific or low affinity DENV antibodies developed in the primary infection (8). There are many hypotheses on how this occurs and no resolutive conclusions have been found so far.

Despite many efforts, there are no universal vaccine nor treatments available against dengue. In 2019, the FDA approved the first dengue vaccine, CYD-TDV developed by Sanofi Pasteur (9). CYD-TDV is a tetravalent live-attenuated vaccine and is currently available in 20 countries in Asia, Latin America, and Australia^1^. The vaccine is successful at preventing severe infection in previously infected individuals but increases the risk of severe disease in dengue-naïve (seronegative) individuals^2^. Consequently, the WHO Global Advisory Committee on Vaccine Safety (GACVS) concluded that individuals who are seronegative should not be vaccinated with CYD-TDV^3^. Therefore, a universal vaccine is still needed to prevent infection, in particular in children whom are more likely to be seronegative. **Table 1** reports CYD-TDV and the additional dengue vaccine and therapeutic candidates. Two other tetravalent live-attenuated vaccines, developed by U.S. National Institute of Health (NIH), Butantan and Takeda, are currently under evaluation in Phase III trials (10, 11). Other dengue vaccine candidates that are currently in clinical development are: D1ME100 DNA vaccine (US Naval Medical Research Center), recombinant DEN-80E (Hawaii Biotech Inc./Merck), and LAV Delta 30 (NIAID/Butantan) (12).

**Table 1:** Dengue vaccines and therapeutics.

Name	Type	Development status	Reference
CYD-TDV	Vaccine	On the market	(9)
TDV	Vaccine	Clinical development	(10)
TV003/TV005	Vaccine	Clinical development	(11)
D1ME100	Vaccine	Clinical development	(12)
DEN-80E	Vaccine	Clinical development	(12)
LAV Delta 30	Vaccine	Clinical development	(12)
Celgosivir	Therapeutic	Failed (ineffective)	(13)
Balapiravir	Therapeutic	Failed (ineffective)	(14)

Table reports name, type, status of development and reference of the dengue vaccines or therapeutics available, in development or attempted to develop.

Although several antiviral therapeutic candidates have been developed, to date none have been effective in treating treat dengue infection (15). The antiviral, Celgosivir (a host α-glucosidase inhibitor) and Balapiravir (a nucleoside analog) were developed for Hepatitis C but were found ineffective against dengue in clinical trials (13, 14). Currently, treatment is solely based on supportive therapy^4^ such as analgesics, fluid replacement and bed rest. The implementation of dengue therapeutics would have a major impact on health and economy worldwide. Breakthroughs in computational analyses and artificial intelligence (AI) are promising approaches to advance and accelerate immunotherapeutic discovery.

## Dengue Genomic Studies Provide Insights on Virus Phylogeny, Pathogenesis and Diagnosis, With Implications in Vaccine Design and Therapeutic Discovery

Dengue virus is a single-stranded RNA positive virus with a 10.7 kb genome. It encodes for three structural proteins: envelope (E), membrane (M), and capsid (C), present in the mature virion (16) and seven non-structural (NS) proteins involved in the intracellular replication phases (NS1, NS2A, NS2B, NS3, NS4A, NS4B, NS5) (17–20). The dengue classification is serologically-based, specifically on the antigenicity of the protein E protein (21). Over the past three decades, as whole genome sequencing and analysis technologies became increasingly affordable and available, many laboratories have started to sequence dengue routinely directly from patient samples generating a large amount of genomic data. Whole-genome studies have led to a better understanding of the genetic composition and diversity of the virus. To date, only DENV1-4 genomes have been sequenced and uploaded onto the NCBI database and the DENV-5 genome sequence has been not reported.

DENV1-4 share 65-70% of amino acid sequence similarity (22). Recent genomic analyses have led to further subclassification of dengue serotypes into distinct genotypes which vary from 6 to 8% (23–26). The NCBI Nucleotide database currently contains 1896, 1472, 925 and 210 complete genome entries for DENV-1, DENV-2, DENV-3 and DENV-4, respectively.

Several genomic studies have determined that there is a strong correlation between specific dengue genotypes and disease severity indicating that differences in genetic sequences that may contribute to increased replication and transmission. For example, it was shown that the Southeast Asian DENV-2 genotype, which caused dengue hemorrhagic fever epidemics, outcompeted the American DENV-2 genotype (which caused only dengue fever) in human dendritic cells and in *Aedes aegypti* mosquitoes (27). This difference in competition correlated with changes in amino acid composition of residue 390 of protein E domain III, the portion of E containing residues which interact with host cell that determine tropism and virulence (28). Leitmeyer and colleagues discovered that the strains causing hemorrhagic fever had an asparagine in position 390, whereas the strains causing fever only had aspartic acid amino acid (29), and hypothesized that this difference could be at least partially responsible for the difference in virulence of the two genotypes.

**Figure 1:**
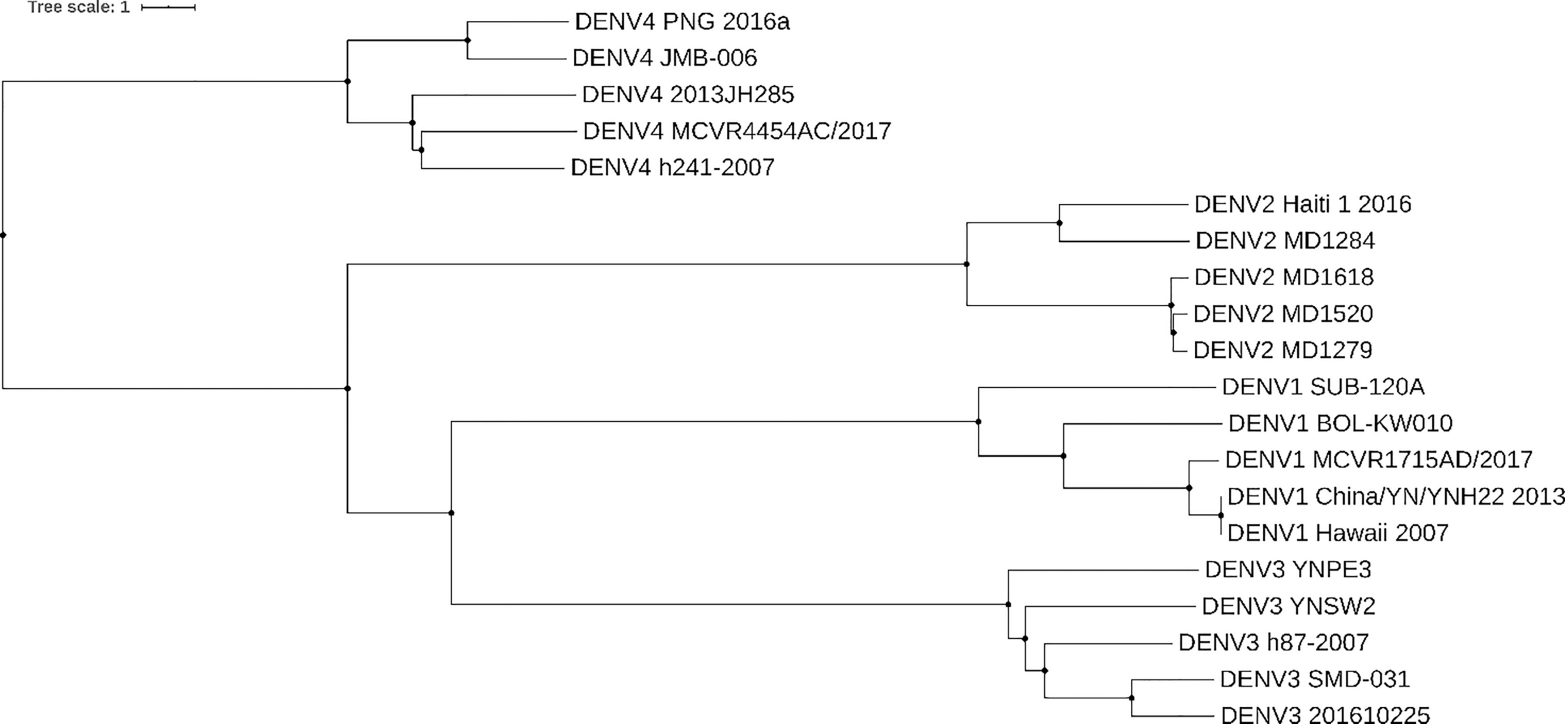
Dengue phylogenetic tree. Neighbor-joining phylogenetic tree based on 5 different isolates of dengue from serotypes DENV-1, DENV-2, DENV-3, and DENV-4. Each taxon indicates a single dengue genome and is labeled with the serotype and isolate name. Genomes were collected from NCBI Nucleotide database. Tree was built with neighbor-joining clustering method using Jalview (30) and visualized with iTOL (https://itol.embl.de/). Source of sequences: for DENV-1 Genbank accession numbers KY057373.1, JQ675358.1, MH891771.1, KY672944.1, MK506262.1, for DENV-2 Genbank accession numbers KX702403.1, FM210244.2, FM210246.2, FM210245.2, FM210242.2, for DENV-3 Genbank accession numbers MF370226.1, KR296744.1, MK506265.1, MH823209.1, KY863456.1, for DENV-4 Genbank accession numbers MH382789.1, MH823210.1, MG601754.1, MH891769.1, MK506266.1.

Whole genome studies have been crucial in constructing phylogenies and epidemiological history of dengue. **Figure 1** shows a phylogenetic tree constructed based on genome nucleotide sequences of different dengue isolates of DENV 1-4 built with neighbor-joining clustering method using Jalview (30) and visualized with iTOL^5^. The tree shows how the different serotypes are phylogenetically related. In agreement with the tree in (31), our tree shows that DENV-4 is the most distantly related serotype. It’s possible to speculate that this is due to greater evolutionary pressure on this serotype, which probably separately emerged into a human urban transmission cycle 500 years ago in either Asia or Africa (32, 33).

Phylogenies have been used to estimate nucleotide substitution rates. Based on analyses, dengue appeared approximately 1000 years ago and was originally sylvatic (transmitted between wild animals and vector). The zoonotic transfer occurred between 125 and 320 years ago, and during the past century, dengue has reached the current global serotype diversity, which has correlated with the population growth and mass transport (24, 33–35). Using phylogenetic studies, intra-host variation was also investigated. It was observed that both the structural and non-structural proteins can vary within the same host and within different hosts/vectors, and was postulated that this variation can influence viral fitness and disease pathogenesis (36–38). Recent genomic studies have led to the development of reverse transcription (RT)-PCR-based diagnostics against the NS1, E, prM and NS5 gene sequences for diagnosis, serotype identification and sequencing for surveillance purposes (39, 40).

**Figure 2:**
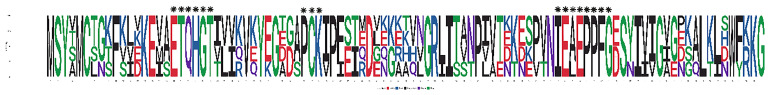
Sequence LOGO of amino acid sequences of dengue protein E domain III. The sequence LOGO shows which amino acids are the most conserved residues among the sequences of domain III of protein E among isolates belonging to different dengue serotypes. Sequences used for this sequence LOGO were obtained from Protein Data Bank (PDB), codes 3IRC for DENV-1, 1TG8 for DENV-2, 4ALA for DENV-4 and 2H0P for DENV-4. Letter height is proportional to amino acid frequencies. The areas of amino acid conservation are marked by black asterisks above the sequence LOGO.

The large quantity of genomic sequence data can be leveraged in other scientific fields such as therapeutic discovery and vaccine design. As genotype sequence has been correlated with virulence, it would be rational to immunize animals with the more virulent dengue virus form resulting in antibodies that target the most virulent variants of the antigen and develop appropriate immunotherapies for the treatment of severe dengue infection. Analysis of genomic data would also be informative for vaccine design. A previous study identified HIV antigen signatures associated with broadly neutralizing antibody-mediated viral neutralization, and used those signatures to rationally design an immunogen eliciting broadly neutralizing antibodies (41). Considering the availability of data about neutralizing antibodies, this approach is feasible for the development of neutralizing antibodies against dengue. Moreover, genomic analysis allows the identification of highly conserved antigens and sub-regions (**Figure 2**), to rationally design vaccines eliciting broadly reactive antibodies, produce broadly neutralizing antibodies for treatment or predict the range of reactivity of an antibody from its epitope.

## Antibodies as Diagnostic Tools and Therapeutics for Dengue

Due to a lack of an effective therapeutic for dengue, several immunological studies have been performed in the effort to discover therapeutic antibodies. Although the generation of antibodies against primary dengue virus is standard, it has been shown that antibodies elicited in the primary infection may enhance the severity of the disease during reinfection by a heterogeneous serotype due to loosely binding anti-DENV antibodies binding Fc-receptor-bearing cells through a method called antibody-dependent enhancement (ADE) (42). Also DENV-specific antibodies can be serotype-specific (43) posing a challenge to the discovery of cross reactive, effective, and universal therapeutics. Fc engineering methods and innovative approaches for the discovery of cross-reactive antibodies may tackle these issues and pave the way for the generation of non-ADE cross-reactive antibodies.

Dengue antigens such as protein E, protein M, protein C, NS1, NS3 and NS5 elicit serum antibodies upon infection (44, 45) and have been used for diagnosis of dengue fever. The diagnosis is based on serological detection of protein E-specific antibodies, together with the detection of the NS1 antigen and molecular detection of viral nucleic acid (46). The hemagglutination inhibition (HI) test is based on the ability of dengue antibodies in sera to inhibit red blood cell agglutination (47). IgM-specific DENV antibodies can be measured using a sandwich capture ELISA (MAC-ELISA) (48). An IgA-specific immunoassay “IgA RT” is also used for diagnosis as a lateral flow test (49). The detection of NS1 antigen from serum can also be detected by several rapid diagnostic ELISA kits (50).

Protein E is the main immunodominant antigen on the viral surface (51), and is the only protein responsible for viral attachment to a broad variety of host cell receptors present on dendritic cells, macrophages, hepatocytes, and monocytes (52–56). Protein E has three domains (EDI–III). Domain III (EDIII), which is the functional portion of protein E, interacts with host receptors and has been reported to be a target for broadly neutralizing antibodies (57–61). Therefore, EDIII is a highly promising antigen to use in studies for the discovery of broadly neutralizing antibodies as dengue therapeutics. Other cross-reactive dengue mAbs target NS1 and can activate complement against infected cells (62). The antibody-eliciting dengue antigens (M, NS3, NS5, C) are unlikely to be good candidates as protein M often elicits antibodies that are poorly or non-neutralizing and enhance ADE (42). NS3 and NS5 are only expressed by the virus intracellularly and do not encounter circulating antibodies (63). Protein C is only exposed within the virion and therefore antibodies would not be able to neutralize this protein (64, 65). **Table 2** summarizes the dengue monoclonal antibodies that have been reported on the NCBI PubMed database to date.

**Table 2:** Dengue mAbs reported in literature and main features.

Name	Specificity	Host of isolation	Antigen	Activity	Reference
9F12	DENV1-4	Mouse	EDIII	Neutralizing	(57)
3E31	DENV1-4	Mouse	EDIII	Neutralizing	(66)
M366.6	DENV1-4	Human	EDIII	Neutralizing	(59)
1A1-D2	DENV1-3	Mouse	EDIII	Neutralizing	(67)
M360.6	DENV1-4	Human	EDIII	Neutralizing	(59)
2H12	DENV1-4	Mouse	EDIII	Neutralizing	(60)
4E11	DENV1-4	Mouse	EDIII	Neutralizing	(68)
Ab513	DENV1-4	Engineered	EDIII	Neutralizing	(61)
SIgN-3C	DENV1-4	Human	EDI, II, III	Neutralizing	(69)
2E8	DENV1-4	Mouse	NS1	Complement activator	(62)
2A10G6	DENV1-4	Mouse	EDII	Neutralizing	(70)
1C19	DENV1-4	Human	EDII	Neutralizing	(71)
d448	DENV1-4	Macaque	EDII	Neutralizing	(58)
J8	DENV1-4	Human	EDI	Neutralizing	(72)
J9	DENV1-4	Human	EDI	Neutralizing	(72)
EDE1 C10	DENV1-4	Human	E dimer	Neutralizing	(73)
EDE1 C8	DENV1-4	Human	E dimer	Neutralizing	(73)
EDE2 A11	DENV1-4	Human	E dimer	Neutralizing	(73)
EDE2 B7	DENV1-4	Human	E dimer	Neutralizing	(73)
D23-1B3B9	DENV1-4	Human	EDII	Neutralizing	(74)

Table reports the dengue mAbs that, to date, have been reported on NCBI Pubmed. Name, serotype specificity, host of isolation, antigen, protective activity and reference are showed for each antibody.

## Large-Scale Immune Repertoire Sequencing to Study the Dengue Immune Response

In the last two decades, the development of next generation sequencing (NGS) technologies for high-throughput sequencing (75), has revolutionized genomics research. In the field of immunology, NGS has broadened the landscape of information on immune responses against certain antigens (76) and has generated a more comprehensive study of immune repertoires (collection of B- and T-cell receptors in an individual). Several high-throughput sequencing approaches have been used for large-scale sequencing of antibody repertoires, including approaches for deep-sequencing of heavy and light chains, CDR3 sequences generated from bulk RNA or genomic DNA (77–79), single cell barcoding (80) and linkage-PCR (81), and single-cell transcriptome sequencing (82).

Large-scale immune repertoire sequencing approaches have been used to elucidate the dengue immune response. Parameswaran and colleagues showed that patients with acute dengue infection have increased B-cell clone expansion and convergent dengue-specific antibody (CDR3) signatures in different patients (77). From our immune repertoire analysis of dengue-infected patient datasets, we have also found that convalescent (7–47 days post symptom onset) and post convalescent patients (180 days post symptom onset) had very similar immune repertoires demonstrating that dengue may leave a strong immune imprint on circulating antibodies long after the disease passes (Horst et al., data not shown). Smith and colleagues showed that the immune repertoire of subjects vaccinated with a live-attenuated vaccine from the National Institute of Health and subjects that were naturally infected have similar antigen specificity, serotype specificity and neutralizing activity (83). Additionally, Appanna and colleagues demonstrated that plasmablast and memory B-cell formation after DENV re-infection involves clonally distinct B cells (84). It was also demonstrated that distinct dengue-infected individuals develop variable titers of antibodies that recognize a specific neutralizing epitope (85). Two recent studies identified and characterized the functional activity and B-cell lineage of broadly neutralizing dengue antibodies (72) and demonstrated that activated, clonally expanded T cells that become committed to the dengue repertoire have a unique transcriptional signature (86). More specifically, this last study applied single-cell RNA sequencing on T-cell receptor (TCR) immune repertoires. This technology accurately reveals transcriptomes of single cells, allowing the identification of transcriptional signatures of different genes in single cells, and could be potentially applied to B-cell receptor (BCR) repertoires to obtain libraries which include paired heavy- and light-chain antibody sequences transcribed in the same single cell to be used for antibody repertoire studies. These studies suggest that dengue infection produces a specific fingerprint on the immune repertoire.

## Analysis of Adaptive Immune Receptor Repertoires Sequencing Data With Machine Learning Methods

Machine leaning (ML) is a computer science discipline and a subset of AI focused on teaching a computer to perform tasks with specific goals without explicitly programming the rules on how to perform this task (87). It allows computers to make predictions based on patterns from data sources, and differently from traditional software, it doesn’t require a specific set of instructions to perform tasks. It includes supervised, unsupervised and reinforcement learning (88). In supervised learning, the computer is provided with labeled training data with the correct responses (targets) and based on this, the algorithm extracts general principles from observed examples (89) and generalizes to predict the correct output to all possible inputs. In unsupervised learning, the data is input without the target labels and patterns are discovered by categorizing similar inputs together. In reinforcement learning, the computer is told the wrong answer but not how to correct it, therefore it analyzes the possible actions and estimates the statistical relationship between the actions and their possible outcomes (90) until it finds the right answer. Machine learning is currently being used to tackle the high complexity of adaptive immune receptor repertoire sequencing (AIRR-seq) data, generated by high-throughput sequencing of the antibody/BCR and TCR repertoires. Specifically, ML applied on AIRR-seq data is used to (i) study repertoire convergence, which is important for the prediction and manipulation of adaptive immunity (91, 92) and has implications in antibody discovery; (ii) analyze repertoire diversity, which can be used for disease detection and vaccine profiling: Greiff et al. developed a framework for immune repertoire profiling based on unsupervised (hierarchical clustering) and supervised machine learning approaches (support vector machine and feature selection) that analyze diversity indices assigned to immune repertoires, generate diversity profiles and predict the immunological status of patients (93), whereas the algorithm developed by Widrich and colleagues, achieves classification of individuals in disease classes based on their immune repertoire thanks to a machine learning method with deep learning architecture with attention based multiple instance learning: a neural network finds pattens in the sequences of the immune repertoire of individuals, maps them to sequence representations, a pooling function generates a repertoire representation and an output network predicts the disease class of the repertoire (94); (iii) predict the antigen or epitope to which T- and B-cell receptors can bind: Jurtz et al. created NetTCR, a method based on convolutional neural networks trained on TCR CDR3 sequences and binding peptide sequences that can filter out from TCR AIRR-seq data peptides that bind to a certain TCR (95), while the recent work from Mason and colleagues showed that deep learning models can generate *in vitro* antibodies retaining antigen-binding properties (96) and predict antibody-antigen binding (97).

## The Power of Artificial Intelligence in Omics Research: A Tool to Diagnose, Predict Diseases and Discover Novel Therapeutics and Vaccines

Artificial intelligence applications such as machine learning offer great analytical capabilities with great potential in “omics” research (98). Modern AI computational approaches can efficiently analyze large datasets obtained from NGS and interrogate the immune repertoire obtaining insights into its diversity (99), evolution (100), and convergence (99, 101) that could not be previously performed, such as predicting antigen-specific antibodies from large datasets of immune repertoires (97, 99, 102). To date, AI has shown great power in different medical fields: (i) It can be used as a disease predictive tool: an example is the “Immunogram”, a series of biomarkers that predicts through AI if the immune system of a patient will be able to overcome cancer; additional examples of the application of AI as a predictive tool are reported in **Table 3**. To date, AI has been primarily used as predictor in cancer, but it could be applied to other diseases, especially to immune-related diseases such as infectious diseases and autoimmune diseases. (ii) It can be used as a diagnostic tool for autoimmune diseases, for example to diagnose Relapsing Remitting Multiple Sclerosis in patients (108). ML was also recently used to understand the differences in BCR between normal and tumor-affected tissues (109), which could be diagnostically used in oncology. (iii) Studies have shown that AI can be used in the discovery, design of new therapeutics and vaccines. Antibody sequences contained in specific antibody clusters developed by spontaneous clearers of Hepatitis C Virus (HCV) have been used for ML-mediated discovery of two anti-HCV broadly neutralizing antibodies (110). Additionally, the HIV envelope neutralizing antibody signatures have been used to predict antibody neutralizing activity and design a trivalent HIV vaccine (41). ML has also been applied to investigate antigen-antibody structures in order to identify paratope-epitope interaction fingerprints and apply them to engineer dengue-specific antibodies with high affinity (111).

**Table 3:** Studies showing possible uses of AI as a predictive tool.

Reference	Main findings
(103)	AI analysis of spatial tumor immune cell interactions and of different biomarkers can be combined with surgical pathology to predict patient response to cancer treatment
(104)	Introduction of the “Immunogram”, a combination of biomarkers that, collectively, can characterize the immune status of a patient affected by tumor and predict if it is sufficient to fight it
(105)	ML can determine the probability of infiltration of a tumor
(106)	Development of MuPeXI, a ML program that identifies the potential of tumor-specific peptides to become neoepitopes
(107)	Development of a computational tool to identify specific T cell subpopulations in non-small-cell lung cancer patients and predict response to immune checkpoint inhibitors treatment

Table reports the reference and main findings of different studies in which AI has been used as a predictive tool in cancer.

## Computational Tools Can Elucidate the Laws That Shape Immune Repertoires

The immune repertoire is complex and can reach more than 10^13^–10^18^ potential specificities (112). Artificial intelligence has a unique capacity to analyze complex data and find underlying patterns, and offers a unique opportunity to deconvolute this complexity. This can be leveraged to study the diversification of the immune system, the repertoire architecture, antibody evolution, and molecular convergence which have important implications in antibody discovery and vaccine design. Several studies are showing advanced investigational potential of the application of artificial intelligence to immune repertoires and could be highly impactful for investigating the dengue immune response when applied to the dengue immune response.

It has been shown that the diversity of repertoires is not generated randomly but evolves following specific rules, leading to the assumption that immune repertoires are predictable in their evolution. Current studies are using AI to elucidate the laws that control repertoire diversity and evolution. Greiff and colleagues have used support vector machines to identify specific immunogenomic rules which shape the diversity of the repertoire (91) and showed that the repertoire is strongly predetermined throughout B cell development from the genetic background and antigen exposure (113). Another study showed that the VDJ recombination process, through which the diversity of the antibody sequences is generated, is biased towards sequences that are likely to pass functional selection (114). The capability of computational methods to elucidate the laws that determine the diversity and evolution of repertoires in response to infectious diseases has already been demonstrated for HIV infections. Sheng et al. found specific hotspots in anti-HIV B cell receptors which are initially highly variable and lose their mutability over time (115). Vieira et al. showed that this loss of mutability is caused by a 60% greater frequency of mutability losses than gains (116) and Hoehn and colleagues developed a substitution model that represents the decay of the mutation rate of B-cell lineages over time, starting from a known germline sequence (117). These studies demonstrate how the rules that shape immune repertoires are starting to be uncovered and understood.

The use of AI to analyze dengue immune repertoires may be beneficial for discovery and predictive studies. For example, since it was shown that the clonotype diversity contains antigen-associated information of the host immune status (118, 119), it may be possible to analyze the evolution of the immune repertoire of multiple dengue serotype-infected individuals and reconstruct how it evolves during sequential infections by different serotypes. This can be used to better understand how the B cell repertoire develops towards antibodies that neutralize different serotypes following first and secondary infections and elucidate the mechanism of adaptation of our immune system to neutralize different antigens. Another possible use of AI would be the comparison of the immune status of dengue-infected individuals affected and unaffected by hemorrhagic fever to understand which clonotypes are more strongly associated with dengue hemorrhagic fever and rationally design vaccines “guiding” the immune response towards antibodies that do not elicit ADE and predict the probability of an individual to develop hemorrhagic fever during reinfection.

## Artificial Intelligence to Predict Antibody-Antigen Interaction and Antibody Specificity

The previous belief that antibodies can recognize a nearly infinite number of antigens and so the interaction between antibodies and antigens is too high-dimensional to be predicted Landsteiner (120) is now being disproven. There are increasing studies in AI that suggest that prediction is possible. Akbar and colleagues have shown that despite generating an innumerable variety of interactions, the paratope-epitope interactions are actually “written” with a finite vocabulary of interaction motifs which are universal (97). The terms used from this vocabulary are always the same, even in completely unrelated antigen-antibody complexes, and ultimately depend on the sequences. Therefore, it is possible to predict antigen-antibody interaction from the sequences. Researchers have developed machine learning algorithms such as Paraped, that has a deep-learning architecture and can predict the paratope of an antibody (*i.e.* the residues that bind to the antigen) from its sequence (121, 122), or the epitope of a protein to which antibodies will bind starting from the protein’s three-dimensional structural data, such as DiscoTope (123). In particular, the sequence of the CDR3, which is the major site of antigen recognition and determines the clonotype (124), seems to be the most attractive target to make sequence-based antibody specificity predictions (125) as it represents a “signature” for antibody specificity. Future studies that apply AI to dengue could leverage CDR3 sequence signatures when predicting immune repertoire specificity. Moreover, the CDR3 sequences could be a good marker to reconstruct the immunological history of a dengue-infected individual and predict the outcome of dengue fever and other infectious diseases (126).

## Training of Machine Learning Methods With Dengue Antibody Sequences Is Allowed by a Great Number of Publicly Available Datasets

A large amount of data is published and could be used to train machine learning methods. For studies aiming at training machine learning methods with dengue-specific antibody sequences, it is possible to acquire data from a large number of dengue antibody publications and databases, such as the monoclonal antibodies (**Table 2**). DENV specific databases also provide a good resource for monoclonal antibody sequences such as DENV-Ab DB^6^ which includes serotype-specific antibodies. The immune repertoire databases available are in **Table 4**. Parameswaran and colleagues (77) report datasets with thousands of dengue-specific heavy chain antibody sequences isolated from acute, convalescent and post-convalescent dengue patients in Nicaragua. Appanna and colleagues report a dataset of dengue-specific B-cell CDR3 sequences from dengue patients in Singapore (84). Another dengue antibody sequence data source is the work by Huang and colleagues (127), who sequenced the IgG immune repertoire from 15 patients in Taiwan. CDR3 sequences of reported antibodies would play a key role in immune repertoire mining, due to their antigen specificity. Godoy-Lozano and coworkers sequenced the IgG heavy chain variable regions from acute patients in Mexico (128). A great amount of data is also available on dengue antigen sequences and may be used to predict antibody specificity from antigen sequences. From hundreds of dengue genomes reported on the NCBI nucleotide database, it is possible to isolate the sequences of the antigen of interest. Two other databases that contain dengue antigen sequences are the Protein Data Bank (PDB), containing 181 dengue dengue-related entries (129), and Dengue Virus Protein Sequence Database (DENV DB)^7^ a database created by the University of Singapore that reports dengue protein sequences from various sequencing experiments carried out worldwide on numerous isolated dengue strains. Additional databases containing dengue-human and dengue-mosquito protein-protein interactions are DenvInt (130) and VirusMentha^8^. These databases provide information for specific protein-protein dengue interaction patterns and identify the bNAbs that can most potently inhibit interaction of the virus with the host. The application of machine learning on these databases to identify new dengue therapeutic or diagnostic antibodies would present a highly innovative use of AI and may provide a model for a new gold standard in antibody discovery.

**Table 4:** Dengue immune repertoire datasets.

Study reference	Dataset type	Dataset source
(77)	Sequencing of Vh regions from 60 individuals in Nicaragua, including 44 dengue patients, 8 patients with non-dengue fever, 8 healthy individuals	www.antibodymap.org
(84)	Sequencing of heavy and light chain variable regions of single B cells from 12 dengue patients in Singapore	Publication
(127)	IgG immune repertoire sequences from 15 patients in Taiwan	European Nucleotide Archive PRJEB13768
(128)	Vh region high-throughput cDNA sequencing of the peripheral blood IgG B cell compartment of 19 individuals in Mexico during acute phase of infection	BioProject ID PRJNA302665

Table reports study reference, dataset type and source of dengue immune repertoire datasets.

## Artificial Intelligence Combined With High-Throughput Immune Repertoire Sequencing Could Elucidate the Dengue Immune Response and Identify Novel Antibody Therapeutics

Dengue-specific bNAbs that we report in this review (**Table 2**) were discovered by employing traditional antibody discovery methods. It is widely proven that the traditional methods for antibody discovery can hampered by several time- and cost-consuming steps involved in high-throughput screening of immortalized B cells (131) and recombinant antibody libraries (132). Moreover, the traditional techniques for antibody screening can be affected by partial loss of antigen-specific antibody sequences, as cell sorting can be suboptimal and result in a poor separation of the desired cells from the total cell population (133). An increasing number of studies are showing that the discovery of new therapeutics can greatly benefit from the use of AI, which can increase the speed of the steps required for the identification of antibodies and improve the accuracy of novel antibody sequence identification by doing a more comprehensive analysis of the antibodyome (complete sets of antibodies), identifying even rare and underrepresented clones. AI has been demonstrated to be able to bypass the antibody screening step by using high-throughput DNA sequencing of B cells and bioinformatic analysis to mine antigen-specific V gene repertoires (Reddy et al., 2010), identify novel HIV neutralizing antibodies directly from B cell transcript sequences (134, 135), and identify opsonophagocytotic antibodies against *Staphylococcus aureus* from DNA sequencing of plasmablasts, outperforming traditional techniques for antibody library generation like the phage display (136). To our knowledge, AI has not been applied to the discovery of dengue-specific antibody therapeutics. New studies applying AI to dengue could lead to impactful discoveries, by identifying among immune repertoire high-throughput sequencing data new dengue antibodies which can’t be identified by traditional methods, further proving the high potential of AI in drug discovery (137), and studying the structure and evolution of the dengue antibody repertoire. In general, we envision a future in the science of antibody discovery in where computational methods will be implemented as a standard antibody identification tool for all infectious diseases.

## Novel ML Platforms Enable Antigen Specificity Prediction

One possible AI tool that we envision that could be used to identify novel dengue bNAbs from AIRR datasets is immuneML. ImmuneML is a recently developed open-source collaborative ecosystem for the application of ML algorithms on AIRR-seq data (138). The platform provides researchers with the possibility to train different ML algorithms with repertoires labeled with metadata both on the repertoire level (e.g., disease/healthy) or on sequence-level (e.g., antigen bound by an antibody) and use them to classify novel AIRR-seq datasets in terms of repertoire classification (e.g., classify a repertoire in sick or diseased) or sequence classification (e.g., classify which antigen is bound by a B cell receptor sequence). These applications are performed using different ML models which are benchmarked, enabling the user to select the best performing model and apply it to new data. We envision the use of immuneML by training with the aforementioned available dengue repertoire sequencing datasets in **Table 3**, and the sequences of bNAbs in **Table 2**, after performing the bioinformatic steps of annotation with annotation software (e.g. IgBLAST, Cellranger VDJ, which are formats compatible with immuneML) and preprocessing (which can performed on a local machine with programs like R, Python or directly in immuneML), to classify AIRR-seq data generated experimentally with platforms enabling large-scale VDJ sequencing (e.g., 10X Genomics VDJ). The use of immuneML would not only offer the possibility to identify possible novel dengue antibody binders from new AIRR-seq datasets, but also to attempt to classify dengue AIRR-seq datasets with a diagnostic valency (e.g., classify a repertoire in dengue-infected/healthy, or classify which dengue serotype affects a dengue-infected individual).

Additional to the use of immuneML, steps towards the identification of successful antibody candidates may be supported by the implementation of a vocabulary of antibody-antigen interaction motifs. This concept was introduced by (97), who showed that the interaction between antibodies and their cognate antigen is, despite the great diversity of the plethora of antigens recognizable by the humoral immune system, based on a finite vocabulary of universally shared epitope-paratope interaction structural motifs, with the consequence that epitope-paratope interaction can be predicted. One way to leverage this discovery for the identification of novel dengue bNAbs is to explore the interactions between the dengue bNAbs reported in literature (**Table 2**) with their cognate antigen, find the antibody sequence residues involved in the interaction with the respective dengue antigen, and look for identical amino acid patterns in the CDR regions of neo-sequenced antibodies. Another possibility is to look for antibodies that have not identical, but similar sequence patterns to the reported dengue bNAbs. This task may be also performed with a combination of networks or sequence alignments and machine learning models such as recurrent neural networks, random forests and support vector machines (Horst, Natali and Miho, data not shown). Noteworthy, a previous study has shown that dengue infection leaves a specific immune fingerprint which corresponds to specific antibody sequence patterns (77). The use of specific epitope-paratope patterns to search for novel bNAbs also enables the research of antibodies that target specific epitopes that are conserved across different DENV serotypes, in order to increase the chance that they are broad binders.

Considering recent advancements in the application of AI to immune repertoires, it is logical to expect impactful results from the application of AI to deconvolute the dengue immune response, which could provide unprecedented insights into the complexity of dengue adaptive immunity, lead to the discovery of new therapeutics which are missed by traditional antibody discovery methods, and inform vaccine design.

## Conclusion

In the last three decades, dengue has been widely studied, leading to a deep characterization of the immune response, genetics, epidemiology and pathology. However, to date, despite the great efforts that have been made to elucidate it, dengue remains a global public health threat which is dramatically spreading worldwide without any resolutive countermeasure. While the only licensed vaccine is not effective in preventing the spread of the virus and therapeutics are lacking, the discovery of a new vaccine or immunotherapy would have a major impact on global health, improving greatly the current catastrophic scenario. Some therapeutic candidates have been tested clinically, and others are currently in preclinical exploration, but most of them are showing lack of efficacy and none have been approved. Artificial intelligence is a highly innovative and versatile analytical tool that can be applied to large datasets such as immune repertoire sequences. It has been tested widely as a predictive tool in cancer and has begun to be used for understanding dengue and other diseases. The application of artificial intelligence to the dengue immune response has great potential. Future studies that apply artificial intelligence to dengue could lead to impactful findings which could help to elucidate the dengue immune response, improve vaccine and immunotherapeutic design and discover new antibody therapeutics.

## Author Contributions

All authors contributed to the article and approved the submitted version.

## Funding

This work has been funded by the Wellcome Trust Innovator Award to EM number 215840/Z/19/Z.

## Conflict of Interest

Author EM owns shares in the company aiNET GmbH.

The remaining authors declare that the research was conducted in the absence of any commercial or financial relationships that could be construed as a potential conflict of interest.

## Footnotes

1
https://www.who.int/immunization/sage/meetings/2016/april/1_Background_Paper_Dengue_Vaccines_2016_03_17.pdf

2
https://www.who.int/news-room/fact-sheets/detail/dengue-and-severe-dengue

3
https://www.who.int/vaccine_safety/committee/GACVS-StatementonDengvaxia-CYD-TDV/en/

4
https://apps.who.int/iris/bitstream/handle/10665/274315/WER9336.pdf?ua=1

5
https://itol.embl.de/

6
http://denvabdb.bhsai.org/denvabdb/

7
https://proline.bic.nus.edu.sg/denvdb/

8
https://virusmentha.uniroma2.it/
